# A Systematic Review of the Link Between Autism Spectrum Disorder and Acetaminophen: A Mystery to Resolve

**DOI:** 10.7759/cureus.26995

**Published:** 2022-07-18

**Authors:** Farhana Yaqoob Khan, Gargi Kabiraj, Maryam A Ahmed, Mona Adam, Sai Prakash Mannuru, Vaiishnavi Ramesh, Ahmed Shahzad, Phani Chaduvula, Safeera Khan

**Affiliations:** 1 Pathology, California Institute of Behavioral Neurosciences & Psychology, Fairfield, USA; 2 Medicine, California Institute of Behavioral Neurosciences & Psychology, Fairfield, USA; 3 Research, California Institute of Behavioral Neurosciences & Psychology, Fairfield, USA; 4 Internal Medicine, California Institute of Behavioral Neurosciences & Psychology, Fairfield, USA; 5 Family Medicine, California Institute of Behavioral Neurosciences & Psychology, Fairfield, USA; 6 Neurology, California Institute of Behavioral Neurosciences & Psychology, Fairfield, USA; 7 Orthopedics, California Institute of Behavioral Neurosciences & Psychology, Fairfield, USA

**Keywords:** asd, tylenol, autism, autism spectrum disorder, autism spectrum disorders, paracetamol, acetaminophen

## Abstract

The purpose of this study is to review the published papers investigating maternal acetaminophen (AP) use during pregnancy and its effect on the offspring's neurodevelopment, particularly autism spectrum disorders (ASD). Acetaminophen is an over-the-counter analgesic and antipyretic considered safe in pregnancy. Recent studies have found an association between acetaminophen and immune system alterations like asthma and adverse neurodevelopmental outcomes. We used online databases (PubMed/Medline/PubMed Central, Science Direct, and Google Scholar) to search the studies relevant to our topic. We screened the papers by titles, abstracts, and then full-text availability. The screened articles were checked for eligibility using relevant quality assessment tools for each study design, extracting and analyzing the data. We finalized 30 studies after the screening; 14 were ineligible. Our final selection included 16 high-quality papers - 13 prospective cohort studies, two review articles, and one meta-analysis.

We found a wide range of neurodevelopmental outcomes in our data collection. So, we included autism spectrum disorders, intelligent quotient (IQ), attention-deficit/hyperactivity disorder (ADHD), isolated language, attention and executive function, communication, behavior, and psychomotor development. All studies showed an association between acetaminophen use and listed neurodevelopmental outcomes. Long-term use, increased dose, and frequency were associated with a stronger association. We extracted collective evidence from 16 studies suggesting acetaminophen's role in developing adverse neurodevelopmental outcomes. It is urgent to do more research on this association before pregnant women can be cautioned about the precise use of acetaminophen.

## Introduction and background

Autism spectrum disorder (ASD) is a heterogeneous developmental disorder that affects all races, ethnicities, and socioeconomic groups [1]. It is defined as persistent deficits in social interaction, communication, and restricted, repetitive behaviors [2]. Almost one in 160 children have ASD worldwide (WHO). In the United States (US), one in every 54 children is diagnosed with ASD [1,3]. It is four times more common in boys than in girls [1]. In the United States, $11.5 billion- $60.9 billion (2011 US dollars) are spent each year on children with ASD [1]. In addition to medical costs, intensive behavioral interventions cost $40,000-$60,000 per child per year [1,4].

What causes ASD is still unknown, and no treatment is available except for early intervention services that help a child learn important skills [1]. We have learned that ASD is a spectrum, and many factors and causes make a child susceptible to ASD, including environmental, biological, and genetic factors [1]. The genetic role in the development of ASD is well known now [1,5,6]. Children with an ASD sibling are more likely to have ASD than the general population [1,7-12]. Some genetic conditions like fragile X syndrome and tuberous sclerosis have a higher risk [1,13]. Some drugs taken during pregnancy have been linked as well [1]. It is investigated that time before, during, and immediately after pregnancy is critical for the development of ASD [1,14].

Acetaminophen is an over-the-counter analgesic and antipyretic. It is also the most common drug taken during pregnancy. Around 65% of US and >50% of European women take acetaminophen during pregnancy. Recent research shows evidence that if taken during pregnancy, acetaminophen may affect the immune system, increase the risk of asthma, and impair neurodevelopment outcomes like behavior and cognition. It is shown that it has disruptive endocrine properties as well [15].

Dr. Anthony Torres first studied the relationship of ASD with acetaminophen and infection during pregnancy 14 years ago. He hypothesized that in genetically predisposed individuals, antipyretics like acetaminophen alter the brain's immune development, leading to ASD. But that hypothesis has recently been investigated further in studies providing good evidence [15].

Because of the limitation of conducting clinical trials on pregnant women, most medications are not adequately investigated in human pregnancies. The safety profile is not established for many medications [15]. Acetaminophen is the only analgesic and antipyretic considered safe to use during pregnancy, so a strong body of evidence about its potential role in the development of ASD is required. Further investigations and reliable studies are needed to find out the underlying mechanisms so that pregnant women can be cautioned about the precise use of this drug during pregnancy or use it safely if it has no association with developmental disorders. We also need further studies investigating genetic contribution or other potential mechanisms that can impair neurodevelopment in selected people.

This systematic review will analyze and summarize the data investigating the use of acetaminophen during pregnancy and its effects on neurodevelopmental outcomes, including ASD in the offspring. We will also highlight the possible mechanisms investigated already in this association.

## Review

Methods

This systematic review was completed strictly following the Preferred Reporting Items for Systematic Reviews and Meta-Analyses (PRISMA) 2020 checklist [16].

Search Sources and Strategy

To search the relevant studies, we used PubMed/Medline/PubMed Central, Science Direct and Google Scholar. The Medical Subject Heading (MeSH) search strategy used for PubMed is 'autism spectrum disorders OR ASD OR autism OR ( "autism spectrum disorder/anatomy and histology"[Majr] OR "autism spectrum disorder/etiology"[Majr] OR "autism spectrum disorder/genetics"[Majr] OR "autism spectrum disorder/history"[Majr] OR "autism spectrum disorder/metabolism"[Majr] OR "autism spectrum disorder/pathology"[Majr] OR "autism spectrum disorder/physiology"[Majr] OR "autism spectrum disorder/physiopathology"[Majr]) AND acetaminophen OR paracetamol OR Tylenol OR ("acetaminophen/administration and dosage"[Majr] OR "acetaminophen/adverse effects"[Majr] OR "acetaminophen/biosynthesis"[Majr] OR "acetaminophen/etiology"[Majr] OR "acetaminophen/history"[Majr] OR "acetaminophen/metabolism"[Majr] OR "acetaminophen/pharmacokinetics"[Majr] OR "acetaminophen/pharmacology"[Majr] OR "acetaminophen/physiology"[Majr] OR "acetaminophen/poisoning"[Majr] OR "acetaminophen/therapeutic use"[Majr] OR "acetaminophen/toxicity"[Majr])'.

For Science Direct and Google Scholar, regular keywords used were: acetaminophen, paracetamol, autism spectrum disorder/disorders, and autism. Boolean "AND" was used as acetaminophen AND autism spectrum disorders to search the relevant articles on these two databases.

Inclusion and Exclusion Criteria

We used specific inclusion and exclusion criteria to filter the initially identified papers. The papers included were from the past five years to get the recent evidence, published in English, and focused on human populations only. The study population was women in their reproductive years, mainly 16+ and children. All study designs were included to get more evidence about the question. No specific geographical area was considered while searching to get data worldwide.

The grey or unpublished literature and studies about adult males were not included. Animal studies were also excluded.

Screening

Two researchers worked independently to screen the remaining studies by going through titles, abstracts, and then the availability of full texts. For some of the relevant papers, which did not have full texts available, the author was also contacted. While screening, we focused on the studies investigating acetaminophen use in pregnancy and its effects on neurodevelopmental outcomes in children, including ASD, ADHD, IQ, language, and motor development. To further reduce the bias, a third opinion was sought in case of conflict.

Quality Appraisal

We assessed the eligibility of the remaining studies independently by using specific quality assessment tools based on study designs by reading the full texts. The Joanna Briggs Institute (JBI) guidelines were used for cohort studies, the Assessing the Methodological Quality of Systematic Reviews (AMSTAR) checklist for systematic reviews and meta-analysis, and the Scale for the Assessment of Narrative Review Articles (SANRA) checklist for one narrative review article used in this systematic review. Articles meeting at least 70% of the criteria have been included.

Data Collection

Two researchers worked independently to identify and extract the data. Then, the data was compiled in table form. It included the year of publication, country, study design, sample size, study population, exposure assessment tools and methodology, outcome measurement tools, and methodology, confounders or covariates, statistical analyses, the confounding adjustment, and finally, the results of each study describing the main outcome. The outcomes included autism spectrum disorders, intelligent quotient, attention-deficit/hyperactivity disorder, isolated language, attention and executive function, communication, behavior, and psychomotor development.

Results

**Figure 1:**
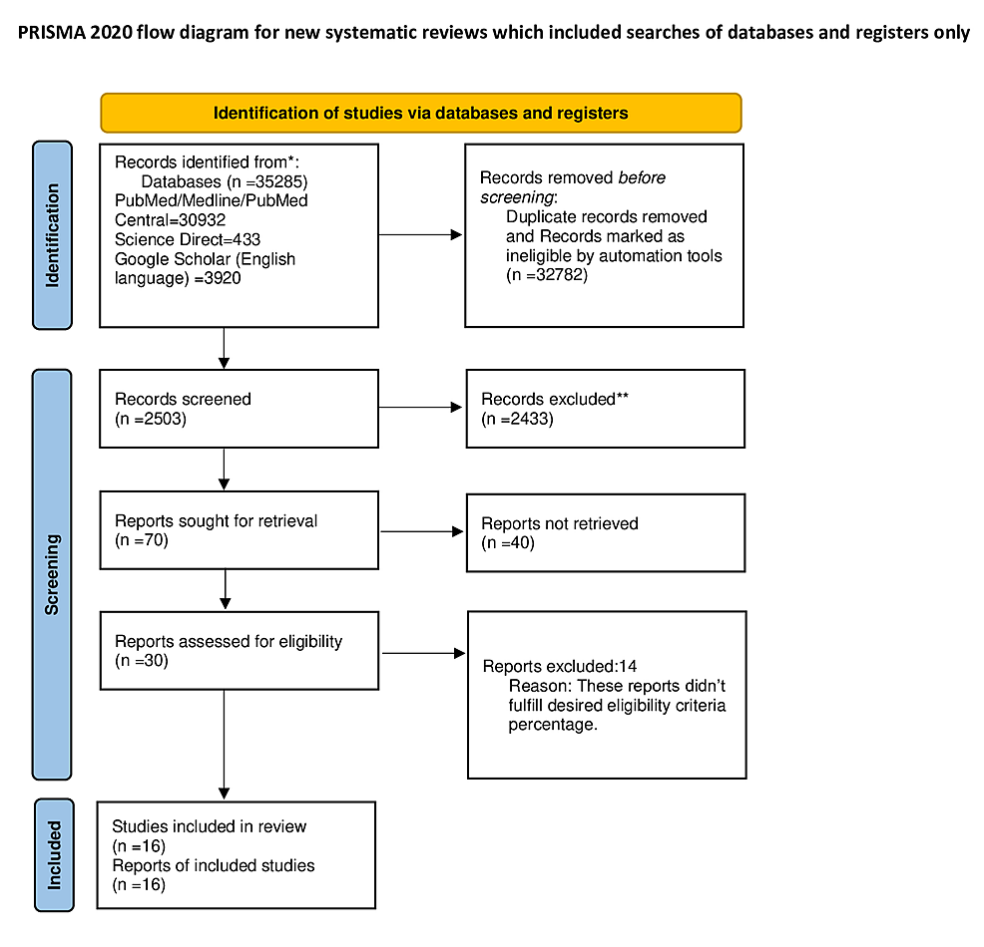
PRISMA 2020 flow diagram PRISMA - Preferred Reporting Items for Systematic Reviews and Meta-Analyses; PM - PubMed; ML - Medline; PMC - PubMed central. *Each database used to identify records is mentioned in the cell. **No automation tools were used in the screening process.

Using the MeSH strategy and keywords mentioned in the method section, we identified a total of 35,285 papers, of which 30,932 were from PubMed, 433 from Science Direct, and 3920 from Google Scholar. We applied the above-mentioned inclusion and exclusion criteria through the automation filter process and removed duplicates manually. We got 2503 studies for screening. After screening through titles and abstracts, 2433 records were excluded. All irrelevant papers not discussing the neurodevelopmental delays and their association with acetaminophen were removed during this process. We then screened 70 papers based on full-text availability. We found 30 full-text papers. These 30 studies were assessed using specific eligibility criteria for each study design, and only 16 highly eligible studies were included for final review. We got 13 prospective cohort studies, one narrative review article, one systematic review, and one meta-analysis in the end. A PRISMA flow diagram 2020 is given in Figure 1.

Discussion

In this systematic review, we will analyze data collected from some of the prospective, longitudinal, and population-based cohort studies and from our review articles to find out the role of acetaminophen in neurodevelopmental disorders.

Does Acetaminophen Affect Neurodevelopment? Evidence from Observational Studies

While analyzing our data, we found out that all the studies were showing similar results. We found collective evidence from thousands of participants in 13 studies that acetaminophen use during pregnancy is associated with several neurodevelopment disorders, including ASD and ADHD. Table 1 shows the summary of exposure and outcome methodology along with study population characteristics.

**Table 1:** A summary of the characteristics of prospective cohorts studies ASD - autism spectrum disorders; AP - acetaminophen; IQ - intelligence quotient; EAS - Emotionality, Activity and Shyness Temperament Questionnaire; BSID - Bayley Scales of Infant Development; MCSA - McCarthy Scales of Children's Abilities; CPSCS - California Preschool Social Competence Scale; CAST - Childhood Autism Spectrum Test; ADHD-DSM-IV - Attention-Deficit/Hyperactivity Disorder Criteria of the Diagnostics and Statistical Manual of Mental Disorders, Fourth Edition Form List; K-CPT - Conner's Kiddie Continuous Performance Test; WPPSI-R - Wechsler Primary and Preschool Scales of Intelligence-Revised; WAIS - Wechsler Adult Intelligence Scale; NPR - Norwegian Patient Registry; ICD-10 - International Classification of Disease; NCE - negative control exposure

Author and year of publication	Study population and sample size	Acetaminophen exposure measurement	Outcome measurement	Statistical analysis
Ji et al. 2020 [17]	Boston Birth Cohort, 996 mother-child pairs were enrolled in the study at birth and were part of this study for the next 10 years in the Boston medical center.	Cord blood samples were collected at birth, and three acetaminophen metabolites were measured in the plasma sample, which is as follows: 1. unchanged acetaminophen, 2. acetaminophen glucuronide, 3. 3-[N-acetyl-l-cysteine-S-yl]-acetaminophen	Data of ASD, ADHD, combined ASD/ADHD, and other developmental disorders (DDs - other behavioral, mental, and neurodevelopmental disorders not related to ASD and ADHD) diagnosed by physicians were collected from the child's medical record.	Odds ratio with 95% confidence intervals.
Tovo-Rodrigues et al. 2018 [18]	2004 Pelotas Birth Cohort (Brazilian population). It included 4231 live births and 3722 children were assessed at age six and 3566 at age 11.	1. Questionnaires investigating perinatal factors were completed at the birth of the children. 2. Interviews during a) perinatal evaluations, and b) follow-up at ages six and 11 years.	Trained psychologists used the Strengths and Difficulties Questionnaires (SDQ) to assess the behavioral symptoms, and standardized scores were assigned to behavioral outcomes.	Crude and adjusted odds ratio. Cutoff values were used for the outcomes.
Bornehag et al. 2018 [19]	SELMA - the Swedish environmental, longitudinal, mother and child, asthma and allergy. It enrolled 754 mother-child pairs at eight to 13 weeks of pregnancy.	1. Maternal interviews 2. Urinary acetaminophen measurement at enrollment, adjusted for creatinine level. 3. AP use was assessed from conception to study entry and the number of tablets taken.	Language development of children at 30 months of age by: a) a nurse (advanced practitioner if required), b) questionnaire on a language scale filled by the parents.	Crude and adjusted odds ratio.
Vlenterie et al. 2016 [20]	Norwegian Mother and Child Cohort Study (MoBa), 51200 mother-child pairs	1. Maternal paper-based questionnaires at gestational weeks 17, 30, and six, 18, 36 months postpartum. 2. AP use is categorized as short-term (1-27 days) and long-term (28 days and more).	1. Maternal interview at child's 18 months of age: psychomotor development was measured by a) Ages and Stages Questionnaire (ASQ), b) objective measurement of child's starting age of unassisted walking, c) The Child Behavior Checklist (CBCL/11/2-5-LDS), d) temperament measurement with EAS.	Odds ratio and numbers needed to harm (NNH) for each outcome.
Avella-Garcia et al. 2016 [21]	Spanish Birth Cohort, 2644 mother-child pairs	1. Maternal interviews by trained evaluators at weeks 12 and 32 of pregnancy. 2. Timing of use was assessed as one month before and during pregnancy 3. Frequency was assessed as never, sporadic and persistent.	At one year: BSID at five years: 1. MCSA 2. CPSCS 3. CAST 4. ADHD(DSM-IV) 5. K-CPT completed by trained psychologists, teachers, and parents.	Relative risk (incidence rate ratios).
Liew et al. 2016 [22]	Danish National Birth Cohort (DNBC, 1996-2002), 1491 mothers and children enrolled in DNBC.	Lifestyle During Pregnancy Study (LDPS) (part of DNBC) provided data about lifestyle factors. Three computer-assisted telephonic interviews at gestational weeks 12, 30, and six months after pregnancy. AP use was categorized as a) ever use, b) never use. AP use in every trimester, every week, total weeks of use and in combination with other medicines were also assessed.	At five years of age: 1. attention function was measured by the Test of Everyday Attention for Children at Five (TEACH-5). 2. Executive function was evaluated by the Behavior Rating Inventory of Executive Function (BRIEF), which was completed by both the parents and the preschool teachers. These tools provided standard test scores for outcomes. The attention and executive function were completed by trained psychologists, who were blinded to exposure status.	Odds ratio.
Parker et al. 2020 [23]	It included 560 mother-child pairs.	Maternal interviews were conducted approximately one year after the delivery. AP use was categorized as short-term (<28 days) and long-term (>28 days). It was measured as any use or no use. AP use before pregnancy was also assessed.	At 6-12 years of age, the child's behavior was evaluated using Child Behavior Checklist (CBC) and Teacher Report Form (TRF). These checklists were completed by mothers and teachers independently.	Unadjusted and adjusted mean differences (MD) and risk ratios (RR).
Arneja et al. 2019 [24]	Ontario Birth Study (OBS), 1200 women enrolled in the study at 11-14 weeks of pregnancy.	Three questionnaires were completed at 12-16, 24-28 weeks of gestation, and 6-10 weeks after the pregnancy (medical and lifestyle data). AP use was assessed three months before pregnancy, at 12-16 weeks and 28-32 weeks. It was categorized as never, early, late, continuous, and never, one per week and more than once per week.	Data about a) child's sex, b) birth weight, c) gestational age at birth was collected from hospital medical charts. It helped evaluate outcomes including preterm birth, low birth weight, and small for gestation age.	Risk ratios.
Ystorm et al. 2017 [25]	Norwegian Mother and Child Cohort Study (MoBa), 112973 children and their parents.	MoBa questionnaires at gestational weeks 18, 30, and six months postpartum. Maternal and paternal assessment of AP use in six months before pregnancy. The use of other medicines was also evaluated.	1. ADHD diagnoses from the Norwegian patient registry (NRP) between 2008 and 2014. 2. Maternal questionnaires when children were six months, one and half years, and three years old.	Hazard's ratio.
liew et al. 2016 [26]	Danish Cohort Study/Danish National Birth Cohort (1996-2002), 1491 mother-child pairs enrolled at six to 12 weeks gestation	Three telephonic interviews at gestational weeks 12, 30, and six months postpartum. AP use is categorized as ever use, trimester-specific use, total weeks of use, never users.	1. Child IQ was assessed at the age of five years by using WPPSI-R by trained psychologists. 2. Maternal IQ was assessed with WAIS and the nonverbal Raven's Standard progression metrics.	Mean differences in child’s IQ using multiple linear regression and scores were calculated.
Leppert et al. 2019 [27]	Avon Longitudinal Study of Parents and Children (ALSPAC). Data collection in ALSPAC started in 1990 and is ongoing. It recruited 7921 mothers. Their genotype data were collected from ALSPAC.	Genotype data were used to estimate the association with 32 maternal early-life exposures (including acetaminophen), and maternal polygenic risk scores were calculated for ADHD, ASD, and schizophrenia. These scores were used to estimate effect sizes.	Questionnaires were used to assess the following factors: 1. maternal lifestyle and behavior (smoking, alcohol, BMI, and maternal age); 2. maternal use of nutritional supplements and medications in the pregnancy (acetaminophen, antidepressants, iron, vitamins, zinc and folic acid); 3. illnesses in the mother (hypertension, diabetes, depression, psoriasis, rheumatism, preeclampsia, infections, or bleeding during pregnancy); 4. factors related to preterm birth, birth weight and cesarean delivery; 5. maternal blood levels of vitamin D, selenium, mercury, cadmium, and lead during the pregnancy.	Odds ratios.
Gervin et al. 2017 [28]	Norwegian Mother and Child Cohort Study (MoBa) A total of 90,000 participants' samples, including a) parental blood samples during pregnancy, b) maternal blood sample and a cord blood sample collected at birth were included.	Three questionnaires. 1. conception to 18 weeks of gestation, 2. 18-30 weeks of gestation, 3. 30 weeks to delivery. Long-term use of AP is categorized as more than 20 days. Trimester of AP use was also recorded. Samples with co-medication history were excluded.	ADHD diagnoses from 2008-2014 were collected from NPR. Specialists made the diagnoses according to ICD-10 guidelines.	DNA methylation analyses were measured with a) microarray preprocessing and quality control, b) differential DNA methylation analysis.
Liew et al. 2019 [29]	Nurses' Health Study II Cohort. The data collected from 1993 to 2005 (1993, 1995, 1997, 1999, 2001, 2003, 2005). This period included two NCE periods, four years before and after the pregnancies and 8856 children were part of the study.	Maternal questionnaires about AP use 1) during the pregnancy: a) regular use (more than two times/week), b) pregnancy status was also recorded at the time of AP intake; 2. before and after the pregnancy: two questionnaires were completed for NCE periods, four years before and after the pregnancy.	Nurse mothers completed a questionnaire in 2013. This included ADHD diagnosis in their biological children and the year they were born. This questionnaire was completed in 2013 when children were around eight years to have a clear diagnosis of ADHD.	Odds ratio with 95% confidence interval.

Ji and co-authors conducted a cohort study in 2020 on 996 mother-infant pairs enrolled in the Boston Birth Cohort [17]. We found recent and better evidence because this study did not rely on interviews and questionnaires, only decreasing the recall bias. Instead, it included cord blood samples collected at birth and measured three acetaminophen metabolites in the plasma. Unchanged acetaminophen was present in all samples. Maternal plasma acetaminophen metabolites collected three days after the delivery was also measured. The children were followed up prospectively from 1998 to 2018. Another aspect of the study providing better evidence is that physician-based diagnoses were used to collect children's medical records. Cord acetaminophen burden was calculated for each outcome. We found that acetaminophen use during pregnancy was more strongly associated with an increased risk of ADHD and ASD than other neurodevelopmental outcomes in a dose-response fashion. After adjusting the confounders, the results were consistent. The interactions were performed between each covariate and cord acetaminophen burden, but these were not significant.

Tovo-Rodrigues and co-authors studied this association to investigate emotional and hyperactivity/inattention symptoms in 4231 children in 2018 in the Brazilian population using data from the 2004 Pelotas Birth Cohort [18]. This study showed that boys exposed to acetaminophen during pregnancy were more likely to have emotional and hyperactivity symptoms at six years of age than at 11 years. The study didn't show this association in girls.

Bornehag and co-authors also studied this association in 2018 using data from the Swedish Environmental, Longitudinal, Mother and child, Asthma and allergy (SELMA), with a sample of 754 mother-child pairs [19]. This study used adjusted urinary acetaminophen concentration in addition to maternal interviews. In addition, it provided stronger evidence by using trained practitioners for language assessment in the offspring at 30 months of age. Acetaminophen was measurable in all urinary samples, and the concentration was compatible with the amount of acetaminophen taken prenatally. Boys had more language delays than girls. But both the number of acetaminophen tablets taken and the urinary acetaminophen concentration were associated with greater language delay in girls but not in boys.

Vlenterie and co-authors conducted a study in 2016 on 51,200 mother-child pairs extracting data from the Norwegian Mother and Child Cohort Study (MoBa) and observed the psychomotor development in children at 18 months [20]. It showed similar results of delayed motor milestones, especially walking, with long-term use of acetaminophen. We also observed a weak association of communication deficits, gross motor impairment, sociability, and shyness with long-term use but not with short-term. This study also estimated the Numbers Needed to Harm (NNH) for motor and communication milestones as 48 and 67, respectively.

Avella-Garcia and co-authors conducted a study in 2016, a subset of the Spanish Birth Cohort Study, on 2644 mothers and children who were evaluated at one and five years [21]. The results were consistent with an increased risk of developing Autism related symptoms in boys with prenatal use of acetaminophen. But the effects on hyperactivity/inattention-related symptoms were the same in all children (boys and girls), and these effects were more strongly associated with increased frequency of use.

A Danish cohort study in 2016 by Liew and co-authors using data from Danish National Birth Cohort (1996-2002) conducted on 1491 mother-child pairs reported acetaminophen adverse effects on child IQ, attention, and executive function, at five years of age [26,22].

We concluded that using acetaminophen during pregnancy was associated with attention problems and poorer executive development in children, especially with a longer duration of acetaminophen use [22]. Only attention deficits were related to first-trimester acetaminophen use, and the effects were not sex-dependent, but parent-related executive deficits were more prevalent in boys. The results were consistent even after adjusting the common confounders of maternal fever, infection, inflammation, pain, or other diseases during the pregnancy [22].

For IQ development, children whose mothers used acetaminophen but didn't have the fever had lower performance IQ, and normal verbal IQ scores compared to kids whose mothers did not have a fever and used acetaminophen [26]. The children whose mothers had a fever and did not use acetaminophen had lower performance and verbal IQ scores at five years. This effect was stronger for first and second-trimester use, and no cumulative effect was seen. Effect modification by child's sex was not seen.

Recently in 2020, Parker and co-authors conducted a study on 560 mother-child pairs using data from the Ontario Birth Study (OBS) to evaluate this association but found a contrast between mother and teacher-reported behavioral problems in children [23]. We found from this data source that scores for behavioral problems in children were higher from maternal data than from the teacher's report giving us an insight to urgently develop more precise and reliable methods or investigations to estimate the role of acetaminophen in developmental disorders.

In addition, Arneja and co-authors studied the effects of acetaminophen on preterm birth, small for gestation age, and low birth weight among children, which are being studied as precursors of neurodevelopmental disorders [24]. Children of mothers who used acetaminophen pre-pregnancy were at higher risk of being small for gestation age and having low birth weight. Increased frequency of acetaminophen use was associated with higher risks of these outcomes. But acetaminophen use during pregnancy did not show any association with these outcomes.

Acetaminophen, an Association With Development of ADHD

There are a few studies investigating acetaminophen increasing the susceptibility of ADHD in children. We included some of these discussing the ADHD and acetaminophen independently of other neurodevelopmental outcomes.

The Norwegian Mother and Child Cohort Study (MoBa) is a large population-based birth cohort study. Ystorm and co-authors extracted data from this study to evaluate the acetaminophen effects on the development of ADHD using 112,973 parents and their children from the study in 2017 [25]. It provided stronger evidence in the form of hazard ratios and a larger sample size than other studies. This study also adjusted data for maternal use before pregnancy, familial risk of ADHD, indications of acetaminophen use, and other potential confounders. We concluded that there is a modest association of any long-term prenatal use of acetaminophen in all trimesters with the development of ADHD. Maternal use before pregnancy had no effect. The use of acetaminophen for fever and infections for 22-28 days also showed a strong association. This study investigated paternal use of acetaminophen before pregnancy, and we found out that it has similar effects on the development of ADHD as maternal use during pregnancy. We should have more studies, including the paternal population, searching for this mystery.

Confounders Have Long Been in the Talk of Acetaminophen Association With Neurodevelopmental Disorders

Recent studies have shown numerous other factors increasing the risk of neurodevelopmental disorders, making acetaminophen's causal relationship problematic. Keeping this in mind, we looked for specific confounders in each study during our data collection. Indications of acetaminophen use, specifically fever, infections, and analgesia during the pregnancy, are considered strong confounders as per date. Table 2 shows all the confounders adjusted in each study:

**Table 2:** A summary of confounders included in all cohort studies AP - acetaminophen; BSID - Bayley Scales of Infant Development; MSCA - McCarthy Scales of Children's Abilities; IQ - intelligence quotient; UTI - urinary tract infection; NSAIDs - non-steroidal anti-inflammatory drugs; ADHD - attention-deficit/hyperactivity disorder; BMI - body mass index; NCE - negative control exposure

Study author	Confounders/covariates Included and adjusted in the study
Avella-Garcia et al. [21].	1. Analgesia, 2. infection, 3. others. The region, child's gender, age at testing, performing psychologist determining the test quality (only for BSID and MSCA), maternal social class, gestational age at birth, maternal IQ, education, chronic illness in mother, fever, or UTI not related to AP use during pregnancy.
Bornehag et al. [19].	Maternal weight, education, smoking, week of enrollment and child's sex.
Liew et al. [26]. (studying child's IQ)	Maternal fever during the pregnancy, infection/inflammation, diseases affecting muscles and joints, child's sex, mother's age at childbirth, parity, parental education index, maternal IQ, smoking and alcohol use during pregnancy, use of NSAIDs during pregnancy.
Ystorm et al. [25].	Parental symptoms of ADHD (measured through self-screening), maternal self-reported smoking and alcohol use during pregnancy, maternal symptoms of anxiety and depression measured at 18- and 30-week gestation, maternal education, age, marital status, BMI at 18-week, parity, birth year centered to 1999.
Vlenterie et al. [20].	Each medical condition and respective medication use during the pregnancy were assessed. Illnesses like pain, fever, and infections were included in the questionnaires. Maternal age at delivery, pre-pregnancy BMI, parity, marital status or cohabiting, education, smoking, alcohol and folic acid use during pregnancy, maternal depression symptoms assessed through The Hopkins Symptoms Checklist, The SCL-5, infections, fever, headache/migraines, pelvic girdle pain, back/neck/abdominal pain, medications (NSAIDs, antiepileptics, antidepressants, opioids, triptans, and benzodiazepines) use during pregnancy.
Gervin et al. [28].	Infant's sex, gestational age at delivery, maternal age, smoking, and alcohol use during pregnancy.
Tovo-Rodrigues et al. [18].	Child's sex, mother's education, age, skin color, parity, smoking and alcohol use during pregnancy, mood issues, infection, pre-pregnancy BMI, NSAIDs use.
Liew et al. [22]. (attention and executive function)	Mother's age at childbirth, parity, parental education index, maternal IQ, maternal anxiety and depression, pre-pregnancy BMI, smoking, and alcohol during pregnancy, use of aspirin or ibuprofen, child's sex, indications of acetaminophen use: 1. fever, 2. infection/inflammation, 3. pain or musculoskeletal disease, 4. mothers who did not experience these symptoms during the pregnancy. Some confounders were adjusted but not included in the final analysis because of minimal effect. These are paternal age, prenatal use of antidepressants, folic acid intake, maternal marital status at the time of interviews.
Arneja et al. [24].	Maternal age, ethnicity, BMI at baseline, education, smoking in three months before pregnancy, fever during pregnancy, paternal smoking in three months before pregnancy, comorbidities (comorbidity index was created), use of other pain relievers like NSAIDs, diclofenac, morphine, oxycodone, and codeine.
Parker et al. [23].	1. Upper respiratory tract infections, 2. headaches, 3. fever, 4. pain/injury maternal age, race, education, marital status, parity/gravidity, smoking and alcohol during pregnancy, paternal age at delivery, pre-pregnancy BMI, infant's sex, medication use for depression and anxiety, other indications of AP use.
Liew et al. [29]. (NCE analysis)	Maternal age at the child's birth, child's birth order, child's birth year, maternal diabetes during the pregnancy, preeclampsia, use of aspirin alone or in combination, other NSAIDs use, AP used in negative control periods (four years before and after the pregnancy), child's sex. Indications of AP use, such as depression, rheumatoid arthritis, and migraine headaches, were included and adjusted. Maternal social factors like income, education of the partner. Lifestyle factors like maternal smoking and alcohol use during the pregnancy.
Ji et al. [17].	Maternal age at delivery, maternal race/ethnicity, mother’s marital status and education level, maternal smoking and alcohol use before or during the pregnancy, history of stress during the pregnancy, maternal BMI, history of breastfeeding, parity, ever use of illicit drugs and maternal fever during the pregnancy. In addition, lead levels in early childhood, sex of the child, birth weight, preterm birth and the delivery type were recorded. Diagnoses of ADHD, anxiety or depression, intrauterine infection, or inflammation in the mother were also included and adjusted. Data for these confounders were collected from questionnaire interviews with mothers by trained research staff and electronic medical records.

All the confounders were adjusted with proper sensitivity analyses in each study, and results were estimated after adjustment. We have included some studies working on the confounding effects of this association.

Liew et al. conducted a prospective cohort study in 2019 using the Nurses' Health Study Cohort II data on 8856 children born in 1993-2005 [29]. This study particularly focused on uncontrolled confounding on acetaminophen association with the development of ADHD by using the negative control exposure (NCE) method. The first evidence this study provided was in line with the previous studies showing an association between prenatal acetaminophen use and ADHD. The NCE analyses showed that this use only during pregnancy was associated with neurodevelopmental effects; NCE analysis pre- and post-pregnancy didn't show any association.

Leppert and co-authors worked on 7921 mothers in 2019 in a population-based cohort study to evaluate the maternal genetic predisposition to the offspring's neurodevelopmental disorders as a confounder of association of these disorders with pregnancy's early life exposures [27]. The maternal genotype data collected from the Avon Longitudinal Study of Parents and Children (ALSPAC) and the polygenic risk scores (PRS-testing of association) were calculated for ADHD, ASD, and schizophrenia with 32 early-life exposures. The data collection started in 1990 and is ongoing, giving us strong evidence explaining that several maternal genetic factors for ADHD are associated with some pregnancy-related exposures, including smoking, maternal depression during pregnancy, prenatal infections, use of acetaminophen in the second half of pregnancy, lower blood levels of mercury and selenium, and higher blood levels of cadmium. We did not find any association between ASD PRS and schizophrenia PRS. This study included almost all the confounders for neurodevelopmental disorders, including acetaminophen. Sensitivity analyses also showed consistent results. These findings suggest that while studying prenatal exposures as causal factors for neurodevelopmental disorders, maternal genetic predisposition should also be considered.

How Does Acetaminophen Affect the Neurodevelopment?

The mechanisms by which acetaminophen contributes to ASD and other neurodevelopmental disorders are unknown. Some studies are discussing about different hypotheses regarding this association.

In a prospective cohort completed in 2017 using data from MoBa, Gervin with co-authors studied DNA methylation differences in children exposed to prenatal acetaminophen and diagnosed with ADHD compared to controls [28]. We concluded from this data source that genetic predisposition and acetaminophen use in combination lead to ADHD. We found that children exposed to acetaminophen long-term 20 or more days during pregnancy had altered DNA methylation compared to controls.

This study used 384 cord blood samples of children at birth, and blood samples of parents were taken during pregnancy and at birth comprising almost all samples from 90,000 participants. We identified several of the top genes linked to ADHD, neural development, and neurotransmission from this data source. By using gene ontology, pathways involved in oxidative stress, neural processes, and olfactory sensory system were also analyzed.

Schultz and co-authors recently in 2021 gave a detailed explanation in a review article about the endocannabinoid system (ECS) and its role in the pathophysiology of ASD [30]. Acetaminophen produces analgesia by acting through the endocannabinoid system. Low levels of endocannabinoids have been found in the blood of ASD people representing low EC tone. In addition, alterations have been found in this system in the brain and immune system of ASD kids. Considering this mechanism, endocannabinoids could be beneficial for ASD kids, so more research is needed to prove the role of ECS in the etiology of ASD.

Bauer and co-authors, in a systematic review in 2018 while working on this association, has included several mechanisms explaining these effects. Possible mechanisms include [15]:

a. Toxic acetaminophen metabolite N-Acetyl-p-benzoquinone imine (NAPQI) formation in excess in the brain affecting neurodevelopment.

b. Acetaminophen effects on increasing oxidative stress, inflammation and affecting immunologic pathways disrupting microglia development and increasing susceptibility of neurodevelopmental disorders.

c. The effects on the endocannabinoid system are important for brain development.

d. Maternal hormones disruption affecting fetus brain development.

e. Cyclooxygenase-2 (COX-2) inhibition.

f. Altered brain-derived neurotrophic factors.

Evidence From Systematic Review and Meta-Analysis

Finally, we included a systematic review to give our findings more evidence. Bauer and co-authors conducted a systematic review in 2018 by including nine publications, prospective/longitudinal, pregnancy, and birth cohort studies. All data sources in this review provided collective evidence of the effects of prenatal use of acetaminophen on different neurodevelopmental outcomes, including ASD, ADHD, and IQ [15].

We included a meta-analysis to make our findings more conclusive of the association. Alemany and co-authors conducted a meta-analysis in six European population-based cohorts in 2021 [31]. This study provided us with a piece of recent evidence from a total of 73,881 mother-child pairs. The use of acetaminophen was also assessed in children up to the age of 18 months, and borderline diagnoses were also used in addition to clinical disorder. After adjusting confounders and indications of use, we concluded that prenatal use of acetaminophen was associated with 19% and 21% more likely to be associated with borderline or clinical ASD and ADHD symptoms, respectively, compared to non-exposed children. Boys had slightly higher odds compared to girls. Neither ASD nor ADHD was associated with postnatal exposure to acetaminophen.

Now we have sufficient data from multiple populations and studies to say that acetaminophen is not as safe as it is considered. The question is why acetaminophen, or any other medication or risk factor, increases the risk of ASD and other neurodevelopmental disorders in certain individuals. We should work on the susceptibility mechanisms through which this risk is increased.

Limitations

While working on this systematic review, we could not find many papers talking about mechanisms of acetaminophen leading to neurodevelopmental disorders in selected groups of individuals. As our exclusion criteria, animal studies were excluded. It is possible that some animal studies could help understand these mechanisms, but still, these studies present a lower level of evidence than human studies. Due to the limitations of conducting clinical trials on pregnant women, drugs like acetaminophen's safety are questioned and need to be explored more in other precise and reliable ways. Although we have significant data in the form of systematic reviews, meta-analysis, and cohort studies providing us strong evidence, it is imperative to work on this area so that pregnant women should be cautioned about precise acetaminophen use during pregnancy. Because of the heterogeneous nature of the final data collected, we could not evaluate a lot of developmental outcomes like ASD.

Acetaminophen's mechanism of action and its relationship to the pathophysiology of disorders, particularly neurodevelopmental disorders, should be explored in an evidence-based manner. If the acetaminophen role is identified, strict avoidance, especially during pregnancy, should be implemented. In that case, further studies on safer analgesic alternatives are imperative.

## Conclusions

We aimed to dins an association between maternal prenatal use of acetaminophen and the development of ASD in offspring. We found heterogeneous data from our selected studies about the effects of acetaminophen on neurodevelopmental outcomes, including ASD. All 16 studies selected in our data showed a consistent association between acetaminophen and adverse neurodevelopmental outcomes. The effects were particularly stronger for attention/hyperactivity symptoms. Long-term acetaminophen use was more strongly associated with the outcomes in a dose-response fashion. Developmental disabilities were more prevalent in boys except for ADHD, where girls were affected equally. We did not see the confounding effects of other risk factors for neurodevelopmental disorders.

Considering this scientific evidence, further studies are urgently needed to strengthen this hypothesis and develop more concrete data collection mechanisms for acetaminophen exposure and outcome measurement. More importantly, further research is needed to investigate the role of genetics, the immune system, and precise mechanisms of actions leading to increased risk in a selected number of individuals. Studies on children's early life acetaminophen exposure and paternal exposure should also be considered. Because of the economic and psychological burden of neurodevelopmental disorders on children, families, and the health care system, pregnant women, should have limited use of this analgesic until proven otherwise. We should arrange systems to educate physicians about limited and precise prescriptions of medications during pregnancy.
